# Content-Seam-Preserving Multi-Alignment Network for Visual-Sensor-Based Image Stitching

**DOI:** 10.3390/s23177488

**Published:** 2023-08-29

**Authors:** Xiaoting Fan, Long Sun, Zhong Zhang, Shuang Liu, Tariq S. Durrani

**Affiliations:** 1 Tianjin Key Laboratory of Wireless Mobile Communications and Power Transmission, Tianjin Normal University, Tianjin 300387, China; 2 School of Electrical and Information Engineering, Tianjin University, Tianjin 300072, China; 3 Department of Electronic and Electrical Engineering, University of Strathclyde, Glasgow G1 1XQ, UK

**Keywords:** visual-sensor-based image stitching, deep homography, mesh warping, content-preserving, edge-assisted

## Abstract

As an important representation of scenes in virtual reality and augmented reality, image stitching aims to generate a panoramic image with a natural field-of-view by stitching multiple images together, which are captured by different visual sensors. Existing deep-learning-based methods for image stitching only conduct a single deep homography to perform image alignment, which may produce inevitable alignment distortions. To address this issue, we propose a content-seam-preserving multi-alignment network (CSPM-Net) for visual-sensor-based image stitching, which could preserve the image content consistency and avoid seam distortions simultaneously. Firstly, a content-preserving deep homography estimation was designed to pre-align the input image pairs and reduce the content inconsistency. Secondly, an edge-assisted mesh warping was conducted to further align the image pairs, where the edge information is introduced to eliminate seam artifacts. Finally, in order to predict the final stitched image accurately, a content consistency loss was designed to preserve the geometric structure of overlapping regions between image pairs, and a seam smoothness loss is proposed to eliminate the edge distortions of image boundaries. Experimental results demonstrated that the proposed image-stitching method can provide favorable stitching results for visual-sensor-based images and outperform other state-of-the-art methods.

## 1. Introduction

With the development of charge-coupled device (CCD) visual sensors and digital visual media, wide-field-of-view panoramic images can provide rich image levels and exquisite image details, which has received great attention over the past few years. As a key multimedia technology to produce high-resolution wide-field-of-view panoramic images, visual-sensor-based image stitching aims at producing multiple images with overlapping regions by rotating the sensors and stitching images by feature matching and image blending. It has played an important role in many multimedia applications, such as photogrammetry [1,2] and remote sensing [3,4]. For instance, some classic image-stitching software products, e.g., Autostitch 1.0 and Adobe Photoshop CS3 [5,6], have promoted computer graphics applications. However, when the image acquisition sensors’ rotation is large or the scene is not coplanar, this may cause obvious artifacts and misalignment. Thus, ensuring that the wide-field-of-view panoramic image has good alignment and naturalness qualities still comprises challenging problems in visual-sensor-based image stitching.

With the rapid recent advances in computer graphics techniques and visual sensor devices, plenty of visual-sensor-based image-stitching approaches have been presented to obtain high-quality resultant stitching images. Traditional visual-sensor-based image-stitching methods include the global alignment method and spatially varying warping method [7]. Global alignment methods utilize invariant local features to match images and establish the mapping relationship by a homography matrix to align the images, such as dual-homography warping [8] and linear transformation stitching [9]. However, some non-overlapping regions may also suffer serious shape distortions. To address this issue, spatially varying warping methods are introduced to divide the image into uniform meshes and optimize the content-based mesh deformation function to obtain the optimal mesh coordinates, including as-projective-as-possible (APAP) [10] and adaptive as-natural-as-possible (AANAP) [11]. However, traditional spatially varying warping methods may cause local structural distortions by using different mesh deformation functions.

In recent years, deep convolutional neural networks (CNNs) have shown their powerful ability in feature matching and correspondence estimation, and some deep visual-sensor-based image-stitching methods have been explored to improve the visual-sensor-based image-stitching performance [12,13,14]. An effective deep image-stitching method aims to estimate the deep mapping relationships between image pairs and blend aligned image pairs to generate natural-looking panoramic images [15]. Nevertheless, many existing visual-sensor-based deep image-stitching methods have two limitations: (1) some methods only depend on estimating a single deep mapping transformation to align image pairs, which sometimes cannot effectively handle large parallax and may twist the global structures of panoramic images; (2) some methods ignore the importance of image content and stitching seams, which easily lead to image content misalignments and discontinuous stitching seams.

In this paper, we propose a content-seam-preserving multi-alignment network (CSPM-Net) for visual-sensor-based image stitching, which ensures an accurate alignment of input image pairs and reduces the content-seam distortions effectively. The major contributions are summarized as follows:

(a) In order to align input image pairs, a content-preserving deep homography estimation was designed to reduce image content inconsistency, and an edge-assisted mesh warping was developed to eliminate stitching seam artifacts.

(b) To ensure accurate image stitching, a content consistency loss was developed to preserve the geometric structures of image pairs. Meanwhile, a seam smoothness loss is proposed to eliminate seam distortions of overlapping regions.

(c) The proposed CSPM-Net was proven to be more effective than state-of-the-art visual-sensor-based image-stitching methods on a real-world database and a synthetic database.

## 2. Related Works

### 2.1. Traditional Image-Stitching Methods

In order to improve the image-stitching performance of visual-sensor-based images, some research works have developed global alignment methods and spatially varying warping methods to conduct visual-sensor-based image stitching recently [16]. Global alignment methods were first presented to stitch multiple images captured from different visual sensors; as a kind of proven and well-adopted technology for estimating global geometric transformation, homography especially is often applied in several visual-sensor-based image stitching tasks [17,18]. For example, Lin et al. [19] introduced a smoothly varying affine field, which was applied to preserve much of the homography ability of image stitching. In most cases, global alignment methods are robust, but often produce discontinuous alignment in the overlapping regions between image pairs.

To solve the model inadequacy of global alignment and further improve image deformation quality, some spatially varying warping methods have been designed recent years [20,21,22,23,24,25]. As a pioneering work, Zaragoza et al. [10] proposed an as-projective-as-possible method, where a moving direct linear transform (DLT) method was designed to adjust the projective warp. In addition, Chen et al. [26] attempted to address the image distortions by a local warp model with a global similarity prior. These methods improve the performance of visual-sensor-based image stitching by adding different constraints to the mesh-grids to realize local alignment. To address the problem of large parallax, Li et al. [27] designed a robust elastic warping method for image stitching, where a Bayesian model was used to remove the incorrect local matches. Similarly, Liao et al. [28] also combined a parametric warp and a mesh-based warp together to stitch images. In addition, Zhang et al. [29] proposed a global optimization method with piecewise rectangular boundaries to realize content-preserving image stitching.

### 2.2. Deep Image-Stitching Methods

Due to the outstanding feature-extraction and feature-matching capability of CNNs, the deep homography method has achieved good performance in many fields recently [30]. In [31], DeTone et al. first proposed a deep homography network for transforming images, where a regression network was applied to estimate the homography parameters and a classification network was used to generate quantized homographies. Shen et al. [32] proposed a parametric alignment based on random sample consensus (RANSAC) to candidate coarse alignment and a non-parametric alignment to predict a dense flow field. In contrast, some unsupervised methods [33] have been presented to solve the homography estimation without true labels. For example, Zhang et al. [34] designed an unsupervised method for estimating deep homography and a triplet loss to optimize the content-aware homography network. Ye et al. [35] designed a deep homography flow to align images, where a low-rank representation block was used to decrease the feature rank and a feature identity loss was applied to optimize the unsupervised process. In addition, Nie et al. [36] introduced a contextual correlation layer in a multi-grid homography, which can represent the transformation in depth-aware images.

Inspired by the idea of deep homography technology, the deep image-stitching method has been proposed to deal with visual-sensor-based images [37,38]. Nie et al. [39] proposed an image-stitching network via global homography to eliminate image artifacts. Considering the importance of edge preservation, an edge-preserving deformation module was trained to produce the image-stitching results [13]. Similarly, Dai et al. [40] also proposed a composition method based on edges for stitching visual-sensor-based images. To address the case of small parallax, Zhao et al. [41] presented a deep homography to estimate the geometric transformation of image pairs. In [15], a deep image rectangling solution was designed to preserve linear and non-linear structures of images. In contrast, an unsupervised image-stitching method [42] was first proposed for image alignment. However, since a single deep homography network is used to align images, these methods may fail in scenes with large parallax. Meanwhile, the importance of image content and stitching seams is often ignored while stitching images, which may cause content distortions and seam discontinuity. To deal with the above challenges, a novel visual-sensor-based image-stitching method based on content-seam-preserving multi-alignment is presented in this paper, which could preserve image content consistency and avoid seam distortions simultaneously.

## 3. Proposed Method

### 3.1. Framework Overview

**Figure 1:**
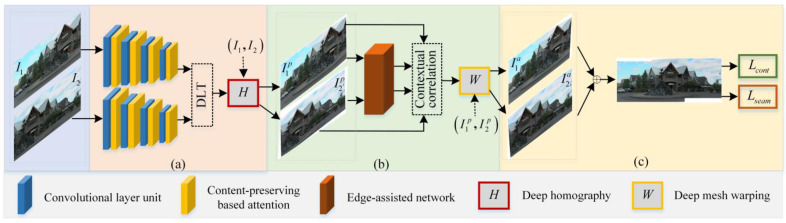
The flowchart of the proposed method. (**a**) Content-preserving deep homography estimation, (**b**) edge-assisted mesh warping, and (**c**) content consistency loss and seam smoothness loss.

The proposed visual-sensor-based image-stitching method via a content-seam-preserving multi-alignment network is described in this section. Figure 1 gives the flowchart of the proposed method, which includes content-preserving deep homography estimation, edge-assisted mesh warping, content consistency loss, and seam smoothness loss. As global and local deep matching features can provide a transformation relationship between image pairs, a content-preserving deep homography is firstly designed to pre-align the input image pairs and reduce content inconsistency. Then, considering that grid-based local transformation can refine the image details, an edge-assisted mesh warping is introduced to further align image pairs and eliminate seam distortions. Finally, in order to preserve the image content and seam information, a content consistency loss is designed to keep the geometric structures of image pairs, and a seam smoothness loss is employed to eliminate seam distortions of overlapping regions. Next, we will introduce the proposed deep image-stitching method in detail.

### 3.2. Content-Preserving Deep Homography Estimation

Image stitching aims to obtain seamless and clear images with a wide field-of-view by composing multiple images with overlapping regions. However, the inconsistency of the object position between the reference and target images easily leads to alignment artifacts and content distortions. In order to obtain high-quality image-stitching results with large parallax, a content-preserving deep homography estimation is constructed to pre-align image pairs and enhance the image content consistency.

For input image pairs, the reference image and target image $\left(I_{1},I_{2}\right)$ both, with a size of U × V, are fed into a symmetric convolutional layer unit to generate the basic visual feature maps of reference and the target branches. Each unit with shared weights consists of two convolutional layers and a max-pooling layer.

**Figure 2:**
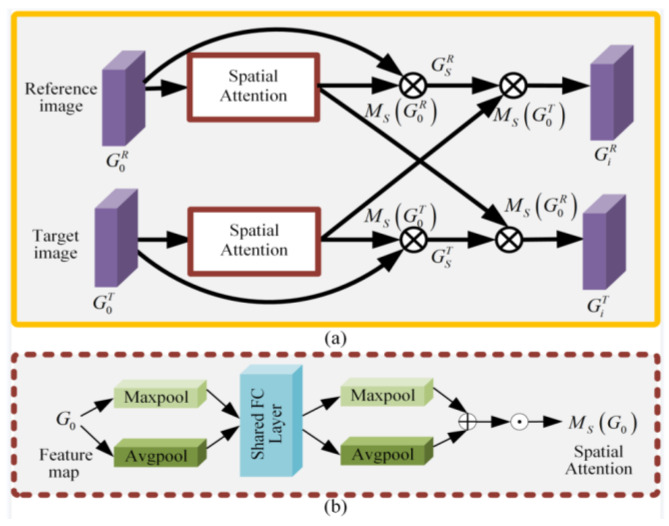
Diagram of content-preserving-based attention module. (**a**) Content-preserving-based attention, where ⊗ denotes elementwise multiplication and (**b**) the spatial attention, where ⊕ denotes elementwise addition, ⊙ denotes the sigmoid function.

Generally speaking, if there is no texture region, repeated patterns, or illumination change in the input image pairs, the homography alignment model is inaccurate due to the insufficient number of matching feature points or uneven feature distribution. In order to align the images and preserve image content accurately, a content-preserving-based attention is introduced into each of the two convolutional layer units to find the correct matching features and eliminate the wrong matching features. The details of the content-preserving-based attention module are shown in Figure 2. For the reference image and target image, considering that the original contents in the non-overlapping regions should be preserved well, the spatial attention is first applied to select the original spatial features of the different contents in a non-overlapping region. Besides, in order to preserve the similar content in the overlapping region, the spatial attention is further injected into each to capture common spatial features of the same content in the overlapping region. Each spatial attention consists of two max-pooling layers, two avg-pooling layers, a shared FC layer, and a sigmoid layer. The output feature maps of each content-preserving-based attention are defined as follows.

(1) $$G_{i}^{R}=G_{S}^{R}⊗M_{s}\left(G_{0}^{T}\right)$$

(2) $$G_{i}^{T}=G_{S}^{T}⊗M_{s}\left(G_{0}^{R}\right)$$

with

(3) $$\left\{\begin{array}{l} G_{s}^{R}=G_{0}^{R}⊗M_{s}\left(G_{0}^{R}\right)\\ G_{s}^{T}=G_{0}^{T}⊗M_{s}\left(G_{0}^{T}\right) \end{array}\right.$$

where *R* and *T* represent the reference image branch and target image branch. $G_{0}^{R}$ and $G_{0}^{T}$ are the input feature maps of the reference and target images. $G_{s}^{R}$ and $G_{s}^{T}$ are the spatialwise feature maps of the reference and target images. $M_{s}\left(\cdot \right)$ is the spatial attention mask. ⊗ is elementwise multiplication. Finally, the DLT method [10] is applied to transform the selected features into the corresponding homography *H*.

### 3.3. Edge-Assisted Deep Mesh Warping

Existing deep image-stitching methods mostly only rely on estimating a single geometric mapping transformation relationship (e.g., homography) to align the reference and target images. However, a single homography cannot align the overlapping regions accurately when the parallax is too large. In addition, if image fusion happens at the overlapping regions with large seam differences, the stitched image may also suffer from seam structure inconsistency artifacts. Thus, an edge-assisted deep mesh warping is proposed to further align image pairs and eliminate seam distortions.

**Figure 3:**
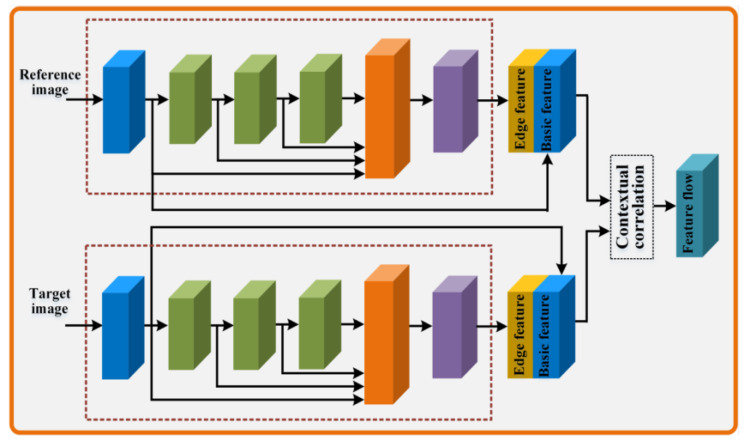
Diagram of edge-assisted network. The blue blocks, green blocks, yellow blocks, and purple blocks mean the convolutional layer, the multi-scale residual block, the upsample layer, and the bottleneck layer, respectively.

In the edge-assisted deep mesh warping, the deep mesh warping is expressed as a multi-grid-mesh-warping problem, in which different homographies are assigned to different pixels in the pre-aligned target image. Specifically, a contextual correlation method [36] is used to take the feature maps of pre-aligned image pairs $\left(I_{1}^{p},I_{2}^{p}\right)$ as the input and outputs a feature flow, which can predict the mesh-grid from the reference image to the target image. Additionally, considering that the edge information of image pairs can be applied as an additional constraint that preserves the seam information, we explored an edge-assisted network that can automatically extract the edge feature maps for eliminating seam distortions. As shown in Figure 3, the edge-assisted network mainly consists of a convolutional layer, three multi-scale residual blocks [43], an upsample layer, and a bottleneck layer. After that, in order to preserve the structurally meaningful edge information of the image pairs, the edge feature maps are further concatenated with the corresponding basic feature maps. Finally, the predicted feature flow of pre-aligned image pairs is computed by the contextual correlation method. The aligned reference and target images $I_{1}^{a}$ and $I_{2}^{a}$ can be expressed as:

(4) $$I_{1}^{a}=W_{con}\left(CCL\left(\left[F_{1c},F_{2c}\right]\right),I_{1}^{P}\right)$$

(5) $$I_{2}^{a}=W_{con}\left(CCL\left(\left[F_{1c},F_{2c}\right]\right),I_{2}^{P}\right)$$

with

(6) $$\left\{\begin{array}{l} F_{1c}=\left[F_{1conv},F_{1edge}\right]\\ F_{2c}=\left[F_{2conv},F_{2edge}\right] \end{array}\right.$$

where $F_{1conv}$ and $F_{2conv}$ are the basic feature maps of pre-aligned image pairs $I_{1}^{p}$ and $I_{2}^{p}$, $F_{1edge}$ and $F_{2edge}$ are the edge feature maps of the pre-aligned image pairs, $F_{1c}$ and $F_{2c}$ are the fusion feature maps, $\left[\cdot ,\cdot \right]$ is the concatenate operation, $CCL\left(\cdot ,\cdot \right)$ is the contextual correlation, and $W_{con}\left(\cdot ,\cdot \right)$ is the deep mesh warping.

### 3.4. Content Consistency Loss and Seam Smoothness Loss

Image stitching aims at producing panoramic images by stitching multiple images with overlapping regions, which are captured from different visual sensors. In order to obtain high-visual-quality stitched images, a content consistency loss and a seam smoothness loss are constructed to reduce the region deformation artifacts and seam discontinuity distortions, respectively. More specifically, to reduce the global alignment and local deformation distortions of image pairs, a content consistency loss is proposed to constrain the shape and position consistency of the objects in the overlapping regions. In addition, to search for the most-accurate stitching seams between image pairs and eliminate linear structures’ distortions, a seam smoothness loss is designed to reduce the visual seam artifacts caused by the local misalignment regions in the image-stitching results.

Content consistency loss: To keep the natural appearance of the stitched image while reducing projective distortions between image pairs, a content consistency loss is designed to encourage the stitched image to have similar pixels and geometry structures to the corresponding ground truth. The content consistency loss *L_cont_* includes the photometric loss *L_photo_* and the structural loss *L_struc_*. To be specific, the photometric loss is applied to minimize the pixel difference between the stitched image and the ground truth, in which the L1-norm is adopted to regularize the photometric consistency. Meanwhile, the structural loss is implemented to encourage the stitched image and the ground truth to have similar feature representations, where the first few convolutional layers of the CNNs are used to provide low-level structural information. The content consistency loss *L_cont_* is defined as:

(7) $$L_{cont}=L_{photo}+L_{struc}$$

with

(8) $$\left\{\begin{array}{l} L_{photo}={\left\|I_{F}-I_{G}\right\|}_{1}\\ L_{struc}=\sum_{i=1}^{2}{\left\|\varphi_{i}\left(I_{F}\right)-\varphi_{i}\left(I_{G}\right)\right\|}_{2}^{2} \end{array}\right.$$

where $I_{F}$ and $I_{G}$ are the final stitched image and the ground truth, respectively, and $\varphi_{i}$ denotes the function of $conv1_{i}$ in the VGG-16 network, in which the receptive field of each pixel in $conv1_{1}$ and $conv1_{2}$ covers a 5 × 5 neighborhood.

Seam smoothness loss: Image stitching must consider both the image content consistency and seam structure continuity. To this end, a seam smoothness loss is designed to reduce seam artifacts in the overlapping regions of the stitched image. Specifically, in order to search for the seam with the minimum differences and further correct the deformation discontinuity of linear structures, the value of each pixel on the object edge for the overlapping regions in the aligned reference image should be closer to that of the aligned target image. Here, we made the edge image of aligned image pairs close to the ground truth edge image of the aligned image pairs. It is worth noting that the curvature formula is applied to obtain the edge image pairs $\left(E_{1G},E_{2G}\right)$ from aligned image pairs $\left(I_{1}^{a},I_{2}^{a}\right)$, which can accurately describe the change in the gradient domain. The seam smoothness loss *L_seam_* is defined as:

(9) $$L_{seam}={\left\|E_{1}-E_{1G}\right\|}_{1}+{\left\|E_{2}-E_{2G}\right\|}_{1}$$

with

(10) $$\left\{\begin{array}{l} E_{1}=E_{net}\left(I_{1}^{a}\right)\\ E_{2}=E_{net}\left(I_{2}^{a}\right)\\ E_{1G}=\mathrm{div}\left(\frac{\nabla_{m}I_{1}^{a}}{\sqrt{1+{\left|\nabla I_{1}^{a}\right|}^{2}}},\frac{\nabla_{n}I_{1}^{a}}{\sqrt{1+{\left|\nabla I_{1}^{a}\right|}^{2}}}\right)\\ E_{2G}=\mathrm{div}\left(\frac{\nabla_{m}I_{2}^{a}}{\sqrt{1+{\left|\nabla I_{2}^{a}\right|}^{2}}},\frac{\nabla_{n}I_{2}^{a}}{\sqrt{1+{\left|\nabla I_{2}^{a}\right|}^{2}}}\right) \end{array}\right.$$

where $E_{1}$ and $E_{2}$ are the edge image of the aligned image pairs, $E_{1G}$ and $E_{2G}$ are the edge images of the ground truth, $E_{net}\left(\cdot \right)$ denotes the edge-assisted network, *m* and *n* represent the horizontal direction and vertical direction, and ∇ and $\mathrm{div}\left(\cdot \right)$ denote the gradient and divergence operations, respectively.

Finally, the content consistency loss *L_cont_* and seam smoothness loss *L_seam_* are combined together; thus, the overall loss *L_All_* is derived as:

(11) $$L_{All}=\alpha L_{cont}+\beta L_{seam}$$

where *α* and *β* are the weights for the content consistency loss and seam smoothness loss.

## 4. Experimental Results and Analysis

### 4.1. Experimental Setup

Implementation details: In order to validate the performance of the proposed deep image-stitching method, we tested the proposed model on two databases. The first one is the real-world database from PTIS [21], where the samples are some challenging image pairs with large parallax. The second is the synthetic database from Warped MS-COCO [39], where the image pairs are without parallax. These two databases contain numerous scenarios, which offer the trained model robustness and generalization. The parameters are shared between the reference and target branches. We used the adaptive moment estimation (ADAM) optimizer method and the initial learning rate 10^−4^, which was divided by 10 after every 10 k iterations. The batch size and momentum were set to 4 and 0.9. The weight *α* and *β* were set to 0.5 and 0.5. After many trials, these were the optimum parameters.

### 4.2. Visual Comparison Evaluation

In this section, we compare the proposed image-stitching method with six other methods, i.e., the APAP method [10], NISwGSP method [23], REW method [24], SPSO method [16], JVCIR method [39], and NC method [41]. The APAP method [10], NISwGSP method [23], REW method [24], and SPSO method [16] are traditional image-stitching methods, while the JVCIR method [39] and NC method [41] are deep image-stitching methods. It is worth noting that the results of the APAP method [10], NISwGSP method [23], REW method [24], and JVCIR method [39] were obtained by running the public source code, and the results of the SPSO method [16] and NC method [41] were obtained by the implementation by us. For space limitations, only the image-stitching results of some typical scenes and the challenging scenes are presented in this section.

**Figure 4:**
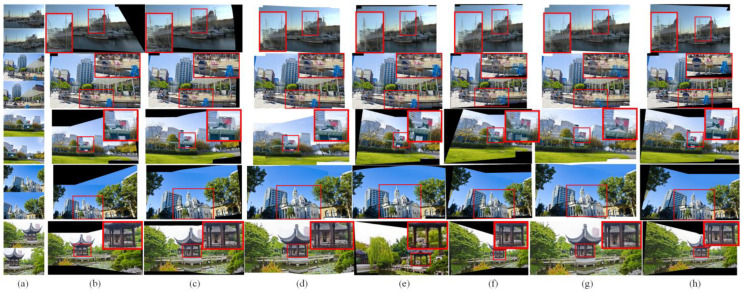
The comparative results for five real-world images. From top to bottom: ship, market, Lawn, church, and pavilion. From left to right: (**a**) the input images, (**b**) APAP method [10], (**c**) NISwGSP method [23], (**d**) REW method [24], (**e**) SPSO method [16], (**f**) JVCIR method [39], (**g**) NC method [41], and (**h**) the proposed method.

Real-world images: Figure 4 shows the visual examples of different image-stitching methods on the PTIS [21] test database. The APAP method [10] obtains natural scenes by utilizing a 2D projective warp with a moving DLT. However, the APAP method [10] degrades the quality of repetitive textures in the final stitched images. For instance, the blue people in the overlapping regions show serious ghosting in the second line of Figure 4b. In contrast, the NISwGSP method [23] preserves the structure of the overlapping regions by the integration of global similarity transformation, but it causes significant artifacts in the non-overlapping regions, such as the street lamp, which has an obvious inclination in the third line of Figure 4c. As given in Figure 4d, the salient objects in the overlapping regions are preserved by the REW method [24], but there is misalignment at the stitching seam in some cases. In addition, the SPSO method [16] is basically able to align image content by the hybrid warping model, but it causes the ghosting of objects in overlapping regions, shown in Figure 4e. Compared with traditional image-stitching methods, the JVCIR method [39] and NC method [41] both show better stitched images in keeping parallax. However, they cannot retain the original proportion of objects due to the limitation of a single deep homography. For example, the pavilion is smaller than the original one in the fifth line of Figure 4f, and the white building is obviously inclined in the fourth line of Figure 4g. In contrast, as shown in Figure 4h, with the help of the integration of content-preserving deep homography and edge-assisted mesh warping, the proposed deep image-stitching method can align the original structure and eliminates the ghosting of the visual-sensor-based images in varying scenes.

**Figure 5:**
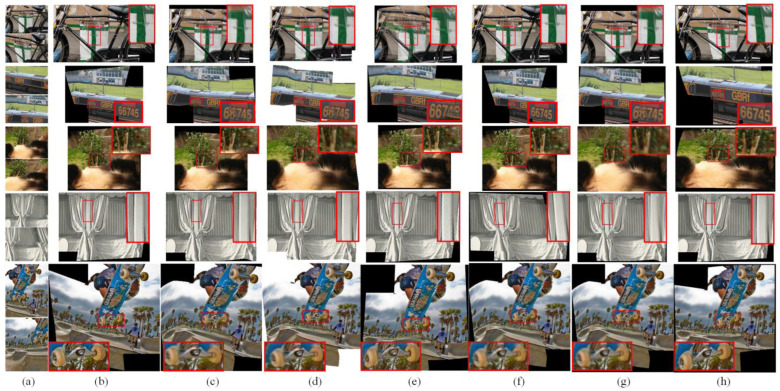
The comparative results for five synthetic images. From top to bottom: bike, train, lawn, curtain, and scooter. From left to right: (**a**) the input images, (**b**) APAP method [10], (**c**) NISwGSP method [23], (**d**) REW method [24], (**e**) SPSO method [16], (**f**) JVCIR method [39], (**g**) NC method [41], and (**h**) the proposed method.

Synthetic images: To further verify the performance of the proposed image-stitching method, we further compared the proposed model with different state-of-the-art models on the synthetic image datasets [39]. Figure 5 shows some challenging images that contain some regions with poor or repetitive textures. The APAP method [10], NISwGSP method [23], and REW method [24] provide natural-looking panoramic images in most cases. However, from the stitched image results, the APAP method [10] enlarges the regions of the scooter in Figure 5b. In addition, the NISwGSP method [23] causes inaccurate alignment in the overlapping regions of the train in the fifth line of Figure 5c, and the REW method [24] contains evident artifacts, such as the middle part of the hair being very fuzzy in the third line of Figure 5d. Similarly, the SPSO method [16] causes distortions in non-overlapping regions. For example, the number on the train is obviously distorted in the second line of Figure 5e. In contrast, for the deep-learning-based image-stitching methods, the JVCIR method [39] obtains desirable image-stitching results with reasonable parallax. However, it fails to align both of the line structures. For example, the curtain is tilted in the fifth line of Figure 5f. Similar to the JVCIR method [39], the NC method [41] also considers the role of deep global homography in image stitching. Therefore, some satisfactory stitched images with fewer parallax distortions are shown in Figure 5g. However, some shape distortions exist in the non-overlapping regions. In contrast, the proposed deep image-stitching method shows superior abilities in avoiding the artifacts, as shown in Figure 5h. This validates the effectiveness of the proposed deep CNN-Net for the image-stitching task.

### 4.3. Quantitative Comparison Evaluation

To further evaluate the performance of the proposed image-stitching method comprehensively, the structural similarity (SSIM) metric [44] and peak-signal-to-noise ratio (PSNR) metric [45] of the overlapping regions were compared between different image-stitching methods. In general, for image stitching, the maximum value of the SSIM metric is 1 and the minimum value of the SSIM metric is 0. Meanwhile, the maximum value of the PSNR metric is infinite and the minimum value of the PSNR metric is 0. The SSIM metrics of the final stitched images from seven different methods are illustrated in Table 1. As can be seen, the SSIM metrics of the APAP method [10], NISwGSP method [23], and REW method [24] are smaller than those from the proposed method, because these traditional image-stitching methods depend on the accuracy of feature detection and matching, which are easily affected by various environments. Unlike these feature-based methods, the performance on the SSIM metric of the JVCIR method [39] and NC method [41] works on dense pixels and achieved high alignment accuracy on some test images. However, the geometric mapping transformation of the JVCIR method [39] and NC method [41] may fail for insufficient feature matching of a single homograph; thus, a few stitched images are bad. In contrast, benefiting from the multi-alignment during the stitching of the images, the proposed method is superior at improving the alignment accuracy of the visual-sensor-based images.

The PSNR metrics of different image-stitching methods are also reported in Table 1. As the baseline spatially varying warping method, it can be seen that the APAP method [10] has the highest PSNR metric, because it showed significant artifacts in the highlighted areas. In addition, the NISwGSP method [23] and REW method [24] provide higher PSNR metrics, as the NISwGSP method [23] yields severe parallax artifacts around the foreground objects, and the REW method [24] ignores the shape-preserving of the overlapping regions. Moreover, the deep-learning-based image-stitching methods, i.e., JVCIR method [39] and NC method [41], exhibit relatively smaller PSNR metrics than the proposed method. Additionally, the proposed visual-sensor-based image stitching model works significantly better than the other methods at aligning the geometry structure and reducing visual artifacts because the proposed network learns more-accurate matching features and mapping relationships, which leads to better robustness.

### 4.4. Ablation Studies

**Figure 6:**
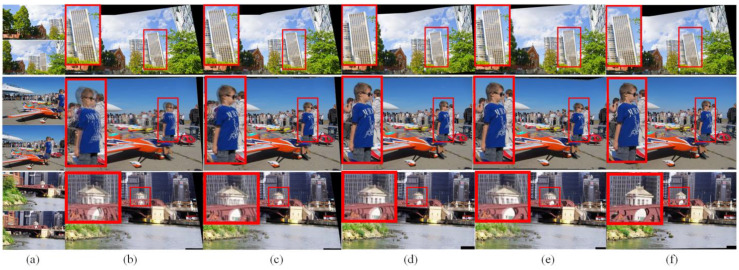
Qualitative comparison results of different cases. From top to bottom: building, plane, bridge. From left to right: (**a**) the input images, (**b**) results without content-preserving homography, (**c**) results without edge-assisted mesh warping, (**d**) results without content consistency loss, (**e**) results without seam smoothness loss, and (**f**) results of the proposed method.

In this section, the ablation studies are conducted to compare the effectiveness of different key components of the proposed model. The qualitative and quantitative evaluation experiments are shown in Figure 6 and Table 2. Specifically, “w/o homography” refers to the proposed method without content-aware deep homography estimation, “w/o warping” refers to the proposed method without edge-assisted mesh warping, and “w/o content” and “w/o seam” refer to the proposed method without content consistency loss and seam smoothness loss.

The qualitative comparison results of different cases are illustrated in Figure 6. From Figure 6b, it can be seen that the case of w/o homography fails to accurately align the reference image and target image, such as the blue boy in the plane image having obvious ghosting and the red bridge in the bridge image is destroyed. Compared with the case of w/o homography, the case of w/o warping has slight visual distortions in Figure 6c. In Figure 6d, the case of w/o content has some content distortions, and the red plane in the final results has content artifacts. In addition, the case of w/o seam in Figure 6e suffers from seam discontinuity, which produces the undesired stitched images. For instance, the tree in the building image has obvious seams. In contrast, the proposed method obtained better stitched results for the visual-sensor-based images in Figure 6f. For example, the original content is preserved well and no visible seams exist in the overlapping regions.

**Figure 7:**
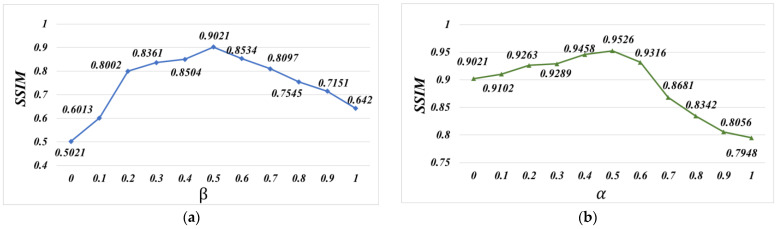
SSIM metric of loss functions with different parameters on 1000 test data. (**a**) The SSIM metric of the loss functions with *α* = 0 and different *β*. (**b**) The SSIM metric of the loss functions with *β* = 0.5 and different *α*.

Figure 7 presents the SSIM metric of the loss functions with different parameters on 1000 test data. In this experiment, α was first set as 0 to yield the best β. As illustrated in Figure 7a, the best SSIM was achieved when β was set as 0.5. Afterwards, β was fixed at 0.5 to search for the appropriate α. From Figure 7b, it is shown that the best SSIM was obtained when α was set as 0.5. The quantitative comparison results of the SSIM metric and PSNR metric are shown in Table 2. From Table 2, the case of w/o homography obtained the worst results, which illustrates that deep homography plays an essential role in aligning images. In addition, the case of w/o content gave worse results than the case of ‘w/o edge’, which validates that the content consistency loss is significant for reducing content artifacts. Furthermore, the case of ‘w/o mesh’ obtains worse results than the proposed method, because of the lack of the multiple alignment operation, and the deep model had some shape and structure distortions. On the other hand, the proposed method outperformed the other cases in stitching the visual-sensor-based images. The quantitative comparison results of the PSNR metric are similar to the trend of the SSIM metric. It can be seen that the proposed deep image-stitching method can reduce significant distortions and avoid intolerable artifacts.

### 4.5. Computational Complexity and Discussions

To demonstrate the efficiency of the proposed method, the average GPU running time of different image-stitching methods is compared in this section. The processing environment was an NVIDIA GeForce GTX 1080Ti GPU. Table 3 shows the running time of different image-stitching methods. For the ship test data, the proposed deep-learning-based method took 0.32 s. For comparison, the APAP method [10] took 0.51 s, the NISwGSP method [23] took 0.5 s, the REW method [24] took 0.95 s, the SPSO method [16] took 0.78 s, the JVCIR method [39] took 0.11 s, and the NC method [41] took 0.12 s. The APAP method [10], NISwGSP method [23], REW method [24], and SPSO method [16] had greater running times in calculating several warping objective energy functions. By comparison, the JVCIR method [39] and NC method [41] took less time than the proposed method, because they only perform an alignment operation to stitch the image pairs. Nevertheless, our method performs the multi-alignment operation, which consists of homography estimation and mesh warping to stitch visual-sensor-based image pairs; thus, it has higher computational complexity.

## 5. Conclusions

In this paper, we proposed a content-seam-preserving multi-alignment network for visual-sensor-based image stitching. Firstly, a content-preserving deep homography estimation was proposed to pre-align image pairs and eliminate image content distortions. Secondly, an edge-assisted mesh warping was conducted to further align image pairs and preserve the valuable stitching seam information. Finally, a content consistency loss was designed to preserve the geometric structure of overlapping regions between image pairs, and a seam smoothness loss was introduced to reduce the seam distortions of image boundaries. The experimental results illustrated that the proposed method outperformed other state-of-the-art traditional and deep learning image-stitching methods for visual-sensor-based images and achieved a 0.9526 SSIM and 26.7321 PSNR on a real-world database and synthetic database. However, the fusion stage of image stitching was not sufficient by simply applying pixel-level fusion methods, which may decrease the performance of image stitching. In the future, the image-stitching performance can be guaranteed by exploring image fusion based on deep feature fusion networks.

## Figures and Tables

**Table 1:** Quantitative comparison between different image-stitching methods. **↑** denotes the larger the value of SSIM and PSNR metrics, the better the quality of image stitching results.

Method	SSIM ↑	PSNR ↑
APAP method [10]	0.8245	20.0453
NISwGSP method [23]	0.8545	20.835
REW method [24]	0.8953	22.3405
SPSO method [16]	0.9198	24.4924
JVCIR method [39]	0.9153	24.5678
NC method [41]	0.9403	26.6984
The proposed method	0.9526	26.7321

**Table 2:** Ablation studies. Data represent the average SSIM and PSNR on 1000 test data. **↑** denotes the larger the value of SSIM and PSNR metrics, the better the quality of image stitching results.

Model	SSIM ↑	PSNR ↑
w/o homography	0.6437	17.394
w/o warping	0.7304	17.659
w/o content	0.8045	18.3921
w/o seam	0.8593	19.0493
The proposed method	0.9153	26.7321

**Table 3:** Running time of different image-stitching methods on ship test data.

Method	Running Time (s)
APAP method [10]	0.51
NISwGSP method [23]	0.5
REW method [24]	0.95
SPSO method [16]	0.78
JVCIR method [39]	0.11
NC method [41]	0.12
The proposed method	0.32

## Data Availability

All datasets used for training and evaluating the performance of our proposed method are publicly available and can be accessed from [21,39].
